# Empfehlungspapier für das körperliche Gruppentraining zur Sturzprävention bei älteren, zu Hause lebenden Menschen: Aktualisierung des Empfehlungspapiers der Bundesinitiative Sturzprävention von 2009

**DOI:** 10.1007/s00391-021-01876-w

**Published:** 2021-04-07

**Authors:** Carl-Philipp Jansen, Michaela Gross, Franziska Kramer-Gmeiner, Ute Blessing, Clemens Becker, Michael Schwenk, die Bundesinitiative Sturzprävention

**Affiliations:** 1 Netzwerk Alternsforschung, Universität Heidelberg, Bergheimer Straße 20, 69115 Heidelberg, Deutschland; 2 Abteilung für Geriatrie und Klinik für Geriatrische Rehabilitation, Robert-Bosch-Krankenhaus Stuttgart, Stuttgart, Deutschland; 3 Deutscher Olympischer Sportbund e. V., Frankfurt am Main, Deutschland

**Keywords:** Sturzprävention, Empfehlungen, Gruppentrainings, Ältere, zu Hause lebende Erwachsene, Umsetzung, Fall prevention, Recommendations, Group programs, Community-dwelling older adults, Implementation

## Abstract

Dieser Beitrag stellt eine Aktualisierung des Empfehlungspapiers der Bundesinitiative Sturzprävention für das körperliche Gruppentraining zur Sturzprävention bei älteren, zu Hause lebenden Menschen aus dem Jahre 2009 unter Berücksichtigung aktueller Evidenz dar. Das aktualisierte Empfehlungspapier zielt darauf ab, die Umsetzung ambulanter Sturzpräventionsgruppen zu fördern sowie konkrete Empfehlungen für deren Einrichtung und Durchführung auszusprechen. Die Empfehlungen beziehen sich auf die Identifikation und Ansprache der Zielgruppe für gruppenbasierte Sturzpräventionsprogramme sowie auf die Programmgestaltung und Qualitätssicherung. Hintergründe zu Finanzierung und Trainer*innen-Ausbildung werden samt einer Auflistung der in Deutschland etablierten Programme ebenfalls dargelegt.

## Abstract (en)

This update of the 2009 recommendations of the German Federal Initiative to Prevent Falls in community-dwelling older persons includes current evidence on state-of-the-art fall prevention group programs and aims to foster their set-up including recommendations on the implementation process. A standardized pathway for identification of the target group as well as guidelines regarding program content and structure, quality assurance, financing, and trainer education are addressed. Established programs being available in Germany are compiled and described briefly.

## 1. Einführung

Die Bundesinitiative Sturzprävention (BIS) ist ein Zusammenschluss von Wissenschaftler*innen und Expert*innen aus dem Bereich der Sturzprävention, von Mitarbeiter*innen von Krankenkassen, Sport‑, Berufs- und Wohlfahrtsverbänden sowie Seniorenorganisationen. Alle beteiligten Verbände oder Einzelpersonen verfügen über vielfältige wissenschaftliche Kenntnisse oder über praktische Erfahrungen in der Umsetzung von Sturzpräventionsmaßnahmen für ältere Menschen am Wohnort. Gemeinsames Ziel der Beteiligten ist es, die Umsetzung von ambulanten Sturzpräventionsgruppen und Einzelangeboten zu fördern.

Die Leitung der BIS liegt derzeit bei Prof. Dr. Clemens Becker, die Geschäftsführung hat der Deutsche Olympische Sportbund (Ute Blessing) übernommen.

## 2. Zielsetzung

Die Mitglieder der BIS haben 2018 beschlossen, gemeinsam das Empfehlungspapier für das körperliche Gruppentraining zur Sturzprävention bei älteren, zu Hause lebenden Menschen aus dem Jahr 2009 in Bezug auf aktuelle Erkenntnisse weiterzuentwickeln sowie ein zusätzliches Empfehlungspapier für das körperliche Training als Einzelangebot für die Zielgruppe zu erarbeiten. Ziel der Konsenspapiere ist es, Empfehlungen für die Einrichtung und Förderung von Gruppen- und Einzelangeboten im ambulanten Bereich zur Sturzprävention auszusprechen. Die BIS hält diese Empfehlungen für wichtig, damit die Einrichtung und Förderung von Sturzprävention für ältere Menschen am Wohnort sinnvoll und wissenschaftlich abgesichert erfolgt, dabei möglichst einheitlich gestaltet wird und nachhaltige Wirkungen erbringen kann.

Ziel des vorliegenden Papiers ist es, die Einrichtung und Förderung von ambulanten Sturzpräventionsangeboten weiter voranzubringen, die den formulierten Qualitätsansprüchen genügen und die flächendeckend umsetzbar sind. Zudem soll es als Aufforderung an zuständige Akteure verstanden werden, einen politischen Diskurs zu diesem Thema zu führen.

Dieses Empfehlungspapier richtet sich auch an Krankenkassen, Verbände, Organisationen und Planungsgremien, die damit unterstützt werden sollen, Auswahlkriterien für eine nachhaltige Förderung zu entwickeln. Dabei wurde beachtet, dass nur Maßnahmen empfohlen werden, bei denen präventive Effekte und eine positive gesundheitsökonomische Bewertung erwartet werden können. Wir freuen uns, wenn es als Beratungsgrundlage für Gremien genutzt wird, die Förderentscheidungen treffen.

Das Papier ersetzt keine wissenschaftlichen Leitlinien und systematischen Reviews. Hier gibt es unseres Erachtens eine ausreichende Anzahl herausragender internationaler wissenschaftlicher Texte von hoher Qualität [17, 18, 37]. Auf der Internetpräsenz des deutschen Zentrums der „Cochrane Collaboration“ findet man Zusammenfassungen aktueller Reviews auf deutscher Sprache.

## 3. Bedeutung der Sturzprävention

Die Auswirkungen des demografischen Wandels in Deutschland sind vielfältig und bereits heute deutlich spürbar. Wir werden in den nächsten Jahren und Jahrzehnten mit Veränderungen konfrontiert werden, die unsere Gesellschaft vor große Herausforderungen stellen. Die durchschnittliche Lebenserwartung steigt kontinuierlich. Bei Frauen liegt sie derzeit bei etwa 83 Jahren, bei Männern bei zirka 78 Jahren [40]. Die steigende Lebenserwartung eröffnet viele Chancen, sie bringt aber auch Probleme mit sich. Eines der Probleme der steigenden Lebenserwartung ist die Zunahme älterer Menschen mit Einschränkungen in sensorischen, kognitiven und körperlichen Bereichen. Dies wird voraussichtlich zu einer Zunahme der absoluten Anzahl an Stürzen führen.

### Stürze sind ein gesellschaftliches Problem

Derzeit ereignen sich in Deutschland jedes Jahr zwischen fünf und sechs Millionen unbeabsichtigter Stürze von älteren Menschen [31]. Mehr als 400.000 Menschen erleiden dabei pro Jahr einen Knochenbruch und werden aufgrund dessen ins Krankenhaus eingewiesen. Die Anzahl der sturzbedingten Hüftfrakturen ist von etwa 120.000 im Jahr 2004 auf mehr als 140.000 im Jahr 2018 gestiegen [22].

Stürze und sturzbedingte Verletzungen gehören derzeit zu den häufigsten Ereignissen, die zu Hause lebende ältere Menschen in ihrer Selbstständigkeit bedrohen. Die individuellen körperlichen und psychischen Folgen eines Sturzes sind oft dramatisch und führen zu einschneidenden Veränderungen. Viele Betroffene entwickeln große Angst, erneut zu stürzen. Sie ziehen sich zurück und verringern ihre körperlichen Aktivitäten, wodurch das Sturzrisiko erneut steigt. Am Ende dieser Negativentwicklung stehen leider sehr häufig der Verlust der Alltagskompetenz und die daraus folgende Pflegebedürftigkeit. So werden die Betroffenen nach einem Sturz häufig in ein Pflegeheim eingewiesen, auch wenn keine Fraktur aufgetreten ist. Stürze ziehen daher oftmals sowohl ein psychisches als auch physisches Trauma nach sich. In vielen Fällen ist das Leben nach einem Sturz nicht mehr dasselbe wie zuvor.

Die häufigen Stürze alter Menschen verursachen hohe sozioökonomische Kosten. Die Kosten für Operationen der durch Stürze verursachten Frakturen, die anschließende Rehabilitationsmaßnahmen der Betroffenen und die häufig aus einem Sturz resultierende Pflegebedürftigkeit der gestürzten älteren Menschen werden von Experten bundesweit auf mehr als 3 Mrd. € pro Jahr geschätzt [22].

Die Sturzursachen sind in vielen Studien untersucht worden [6, 10]. Wichtige Risikofaktoren sind eine nachlassende Gleichgewichtsfähigkeit und eine Reduktion der Muskelkraft. Durch ein kombiniertes Training von Gleichgewicht und Kraft kann das Sturzrisiko deutlich reduziert werden. Dies ist die mit Abstand wirksamste Methode zur Sturzprävention [37]. Neben der Verbesserung der physischen Ressourcen müssen auch die psychosozialen und mentalen Ressourcen im Training angesprochen und verbessert werden. Diese sind ein fester Bestandteil eines guten Gesundheitssporttrainings.

### Sturzprävention für zu Hause lebende Menschen

Es ist seit 2009 in Deutschland gelungen, verschiedene evidenzbasierte Programme für ältere, zu Hause lebende Menschen zu evaluieren und zunehmend zu verbreiten. Dies erscheint von großer Bedeutung, da die Mehrzahl älterer Menschen nach wie vor zu Hause lebt und dies der Präferenz der meisten Menschen für deren zukünftiges Wohnen im Alter entspricht.

Während für ältere Menschen ohne Sturzrisiko zunehmend Mobilitätsangebote vorhanden sind (z. B. Bewegungskurse in Vereinen), besteht für zuhause lebende ältere Personen mit Sturzrisiko ein erhebliches Versorgungsdefizit an gezielten Programmen. Gruppenangebote werden zwar vereinzelt angeboten, eine flächendeckende Einführung ist aber bislang nicht gelungen. Ein aufsuchendes Angebot für Personen mit hohem Sturzrisiko, die besonders von gezielten Trainingsmaßnahmen zur Sturzprävention profitieren, Gruppen aber nicht mehr besuchen können, steht in Deutschland bisher überhaupt nicht zur Verfügung. Hieraus ergibt sich die Notwendigkeit, im ambulanten Bereich weiter zu handeln.

## 4. Zielgruppen für die Sturzprävention in der Gruppe

Der „Steadi“-Algorithmus [4] für das Screening, Assessment und die Intervention wurde ins Deutsche übersetzt und an die nationalen Gegebenheiten angepasst. Dies ermöglicht eine sturzrisikobasierte Zielgruppeneinteilung, die auch ein gewisses Maß an Durchlässigkeit zwischen den Gruppen vorsieht. Dies bedeutet, dass die Zuordnung zu einer der beiden Risikogruppen nicht zwingend die Interventionsmaßnahme vorgibt, sondern individuell entschieden werden muss, ob die Person an einer Gruppenform oder einer individuellen Einzelmaßnahme teilnehmen sollte.

**Figure 1:**
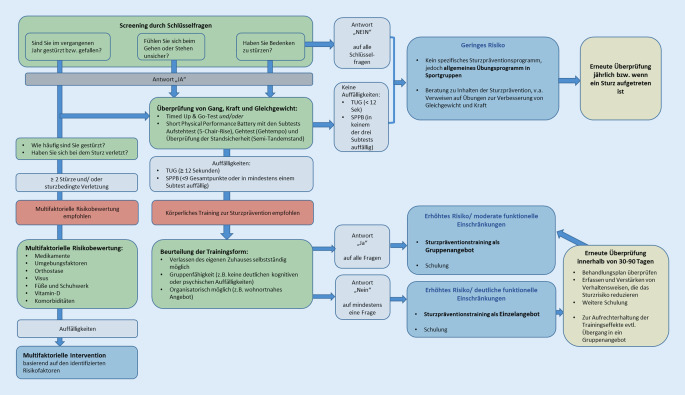

Detaillierte Testkriterien bzw. Cut-off-Werte für die Funktionstests in Anlehnung an den „Steadi“-Algorithmus (Abb. 1) zur Identifikation der Zielgruppen sind untenstehend zu finden.

*Grundsätzlich gilt:* Eine medizinische Voruntersuchung ist prinzipiell nicht erforderlich. Eine Information der Haus- oder Fachärzt*innen jedoch ist ausdrücklich erwünscht.

**Ältere Menschen ohne bzw. mit geringem Sturzrisiko** sind nicht für eine spezifische Intervention vorgesehen, sollten jedoch nach „Steadi“ an einem allgemeinen Übungsprogramm in Sportgruppen teilnehmen, die im Idealfall Übungen zur Verbesserung von Gleichgewicht, Kraft und Koordination beinhalten.

Es ergeben sich folglich zwei Gruppen, für welche eine Teilnahme entweder an einem Gruppensturzpräventionsprogramm oder einem Einzelangebot empfohlen wird:

### Ältere Menschen mit erhöhtem Risiko und moderaten funktionellen Einschränkungen

Bei der Zuordnung zu einem Sturzpräventionsangebot sind sowohl medizinische Diagnosen, ein spezifisches Alter, Pflegegrad oder eine vorliegende demenzielle Erkrankung per se kein Einschluss- oder Ausschlusskriterium, jedoch sollten diese Faktoren eine Gruppenteilnahme noch zulassen. Viele der Teilnehmer*innen dieser Zielgruppe haben Hypertonie, Diabetes mellitus, Arthrose und/oder andere chronische, medikamentös kontrollierbare Erkrankungen. Die meisten Teilnehmenden sind in der Regel über 75 Jahre alt.

#### Einschlusskriterien

- Sturzanamnese (einen oder mehr Stürze in den letzten 12 Monaten)
  *und/oder*
- subjektive Gang- oder Standunsicherheit
  *und/oder*
- Bedenken, zu stürzen.

#### Ausschlusskriterien

- Personen, die keinen Zugang zu Gruppenangeboten haben
  *und/oder*
- Personen, die ihr Zuhause aufgrund von körperlichen oder psychischen Einschränkungen nicht verlassen können oder wollen
  *und/oder*
- Personen, die nicht gruppenfähig sind (z. B. durch ausgeprägte kognitive oder psychische Auffälligkeiten)
  *und/oder*
- Personen, die keine Auffälligkeiten gemäß Timed Up-and-Go Test oder Short Physical Performance Battery haben (gemäß unten dargestellten Testkriterien).

Es wird an dieser Stelle darauf hingewiesen, dass das Vorhandensein eines Pflegegrads kein Ausschlusskriterium darstellt.

### Ältere Menschen mit erhöhtem Risiko und deutlichen funktionellen Einschränkungen

Personen, welche mindestens eines der ersten drei Ausschlusskriterien erfüllen, sollten für die Teilnahme an einem Einzelangebot in Betracht gezogen werden. Nach ca. 30 Tagen sollten diese drei Kriterien erneut geprüft werden, um einen möglichen Übergang in ein Gruppenangebot zu evaluieren.

### Testkriterien zur Identifikation der Zielgruppen

Nach wie vor gibt es keinen „goldenen“ Standard bei den Messverfahren zur Erhebung des Sturzrisikos [2, 34]. Bei der Wahl des Testverfahrens ist es wichtig, darauf zu achten, dass die wichtigsten Bereiche mit dem höchsten Einfluss (Kraft, Gleichgewicht, Gangeinschränkungen) zumindest bei den physischen Ressourcen durch den Test erfasst werden. Dabei gilt es, der großen Heterogenität und den beabsichtigten Zielen bei der Erfassung der funktionellen Leistungsfähigkeit bei älteren Menschen gerecht zu werden. Zum einen sollen die Tests als Screeningverfahren verwendbar sein, zum anderen sollen durch die Intervention bedingte Veränderungen auch erfassbar sein (Veränderungssensitivität), damit sie zur Qualitäts- bzw. Effektkontrolle verwendet werden können. Außerdem ist eine einfache und schnelle Anwendbarkeit von Vorteil.

Gemäß „Steadi“-Algorithmus (Abb. 1) werden im Folgenden verschiedene Testverfahren aufgeführt, die für eine Zuordnung in die entsprechende Zielgruppe herangezogen werden können. Bei der Auswahl der Testverfahren wurden Testgütekriterien sowie ökonomische und praktische Überlegungen berücksichtigt.

#### Modified Timed Up-and-Go (TUG)

Der TUG [29] ist ein Screeningtest zur Erfassung der funktionalen Mobilität und des Sturzrisikos. Als mehrdimensionales Assessment gibt er insbesondere Auskunft über das Vorhandensein von Einschränkungen hinsichtlich Beinkraft, Gang und dynamischer Gleichgewichtsfähigkeit. Aufgrund seiner sehr guten Anwendbarkeit, sehr guten Reliabilität und guten bis sehr guten Validität [5] kann der Einsatz empfohlen werden.

#### Short Physical Performance Battery (SPPB)

Eine Testbatterie, die die wichtigsten motorischen Dimensionen Kraft, Ganggeschwindigkeit und Gleichgewicht abdeckt, ist die *Short Physical Performance Battery (SPPB) *von Guralnik et al. [15, 16]. Diese Testbatterie ist im internationalen Raum zur Kategorisierung des Sturzrisikos weit verbreitet und verfügt über Referenzwerte für unterschiedliche Kohorten [28, 42]. Zudem zeigt die SPPB eine nachweislich gute Reliabilität und Validität [13].

Die Testbatterie beinhaltet:

- einen Chair Rise Test (5 × vom Stuhl aufstehen und hinsetzen),
- einen Test zur Messung der Ganggeschwindigkeit über 4 m,
- einen Gleichgewichtstest mit progressiver Anforderungssteigerung.

Beruhend auf den Testgütekriterien, der Verbreitung und den eingeschlossenen Dimensionen, der Möglichkeit, einen Summenscore zu bilden, sowie auch einzelne Werte in den drei Dimensionen zu erhalten, spricht sich die BIS zur Nutzung dieses Testverfahrens aus.

### Zuteilung potenzieller Teilnehmer*innen in ein Einzel- oder Gruppenangebot

Um ältere Menschen der Zielgruppe mit „erhöhtem Risiko/moderaten funktionellen Einschränkungen“, für welche eine Gruppenintervention empfohlen wird, von den Personen der Zielgruppe „erhöhtes Risiko/deutliche funktionelle Einschränkungen“ abzugrenzen, für die ein Einzelangebot empfohlen wird, können insbesondere Subtests der SPPB-Testbatterie und der TUG verwendet werden (Tab. 1). Eine Beurteilung der besseren Eignung einer Gruppen- oder Einzelmaßnahme sollte nicht nur auf einem einzelnen Testergebnis basieren. Vielmehr ist es ratsam, sich ein umfassendes Bild zu machen, welches Auskunft über das Maß der Einschränkung gibt und damit verbunden die geeignete Interventionsform bestimmen kann.

**Table 1:** 

Test	Einzelprogramme	Gruppenprogramme
TUG (3 m)	>15 s	12–15 s
Gehtempo (4 m Gang)	<0,8 m/s	≥0,8–1,2 m/s
5 Chair Rise	>15 s	12–15 s
Semitandemstand	<10 s	10 s
SPPB-Gesamtscore	<9 Punkte + weitere Einschränkung(en)	<9 Punkte

## 5. Zur Gestaltung der Gruppentrainingsprogramme

Gruppentrainingsprogramme haben sich zur Sturzprävention als effektiv erwiesen [37]. Wichtig dabei ist, dass die Programme regelmäßig mindestens einmal pro Woche, besser jedoch zweimal pro Woche möglichst lange durchgeführt werden [19, 20]. Im Sinne der Selbstbefähigung der Teilnehmer*innen wäre es ideal, die Übungen einmal wöchentlich in der Gruppe sowie ein weiteres Mal selbstständig zu Hause durchzuführen. Dies setzt eine erfolgreiche Befähigung der Teilnehmer*innen zum eigenständigen Trainieren zu Hause voraus.

### Trainingsinhalte/Trainingsempfehlungen

Aktuelle Metaanalysen und systematische Reviews [11, 14, 36, 37, 44] dokumentieren die besondere Effektivität von **Trainingsprogrammen**, welche mehrere Komponenten wie beispielsweise funktionelle Kraft- und Gleichgewichtsübungen sowie Koordinationsübungen beinhalten und kombinieren.

Ein **funktionelles Gleichgewichtstraining** bei Senior*innen sollte sowohl statische Übungen mit einer Verkleinerung der Unterstützungsfläche (bspw. (Semi‑)Tandemstand und Einbeinstand) als auch dynamische und reaktive Übungen beinhalten, in denen der Körper bewusst aus dem Gleichgewicht gebracht wird, wie bspw. beim Tandemgang und verschiedenen Arten von Körperdrehungen (=selbstinduzierte Perturbationen) [37]. Des Weiteren sind Übungen, welche die an der posturalen Kontrolle beteiligten Muskelgruppen des Körpers beanspruchen, wie Fersen- und Zehenstand, sowie Mehrfachaufgaben [33] und Variationen in der Sensorik (z. B. Trainieren auf unebenem Untergrund oder mit geschlossenen Augen) anzustreben [26]. Der Schwierigkeitsgrad der Übungen sollte hierbei individuell an die Funktionsfähigkeit der Teilnehmer*innen angepasst und progressiv gesteigert werden [9, 32, 37]. Darüber hinaus empfiehlt die BIS die Thematisierung psychosozialer Aspekte; insbesondere Sturzangst sollte aufgearbeitet werden, eine Hinführung zum Zusammenhang von Wohlbefinden und körperlicher Aktivität sowie die Berücksichtigung individueller Motive und Barrieren und kognitiver Aspekte ist sinnvoll.

Um funktionelle Alltagsleistungen wie Treppensteigen und Haushaltsarbeiten so lange wie möglich bewältigen zu können, werden zusätzlich zum Gleichgewichtstraining **funktionelle Kraftübungen** empfohlen. Das funktionelle Krafttraining sollte vor allem Übungen zur Steigerung der Kraft der unteren Extremitäten berücksichtigen [1, 35, 37]. Dabei sollte nach einer Aufbauphase eine moderate bis hohe Intensität (60–80 % des maximalen Gewichts, mit dem eine Wiederholung geschafft wird) bei zwei bis drei Wiederholungssätzen gewählt werden. Die Übungsanzahl kann, je nach Leistungsfähigkeit der Teilnehmer*innen, bei sechs bis acht Übungen mit einer jeweiligen Wiederholungsanzahl von zehn bis zwölf Wiederholungen pro Satz liegen. Alleiniges Krafttraining reicht aber nicht aus, um einen signifikanten Rückgang der Sturzraten zu erreichen.

Sichergestellt werden sollte insgesamt, dass die Teilnehmer*innen idealerweise zweimal pro Woche das Übungsprogramm durchführen [45]. Diese könnte ein mindestens einmal wöchentlich stattfindendes, supervidiertes Programm sowie zusätzlich durchgeführte, nichtsupervidierte Übungseinheiten zu Hause beinhalten.

### Mindestdauer

Die BIS empfiehlt vor dem Hintergrund theoretischer Modelle zur langfristigen körperlichen Aktivität bei älteren Erwachsenen [27], prinzipiell langfristige Angebote zu installieren und zu fördern, denn sturzgefährdete Menschen benötigen ein lebensbegleitendes Training, um Stürzen dauerhaft entgegenzuwirken. Zeitlich limitierte Angebote bergen das Risiko, dass die gesteigerte Funktionsfähigkeit der Teilnehmer*innen nach Ablauf der Maßnahme wieder nachlässt und das Sturzrisiko dadurch wieder ansteigt. Falls die Einrichtung von langfristigen Angeboten nicht umsetzbar ist, sollte die Mindestdauer der Interventionen 2–3 Monate betragen, 6 Monate sind anzustreben [37].

### Kurstrainer*innen und Gruppengröße

Bis dato gibt es eine große Breite an verschiedenen Professionen, die zur Anleitung von Gruppentrainingsprogrammen zur Sturzprävention qualifiziert bzw. zugelassen sind: von Gesundheits- (Physiotherapeut*innen) und Sportexpert*innen (Sportlehrer‑/Sportwissenschaftler*innen) bis hin zu ausgebildeten Bewegungstherapeut*innen und Übungsleiter*innen. Eine detaillierte Auflistung der Qualifikationen ist in Kap. 9 dargestellt. In der aktuellen Übersichtsarbeit von Sherrington et al. [37] konnte gezeigt werden, dass die Effektivität der Interventionen nicht davon abhängt, welche der qualifizierenden Berufsausbildungen die Trainer*innen absolviert haben, solange sie eine Setting-spezifische Ausbildung durchlaufen haben.

Um ein sicheres und individuelles Trainieren zu gewährleisten, sollte bei leistungsschwächeren Teilnehmern*innen eine Gruppengröße von bis zu 15 Teilnehmern*innen nicht überschritten werden [1, 9].

Legt man die Ergebnisse von Sherrington [37] zugrunde, zeigt ein intensives, individuell herausforderndes Trainingsprogramm, bestehend aus Gleichgewichts- und funktionellen Übungen (inklusive Kräftigungsübungen), einen besonders hohen Effekt.

## 6. Ansprache der Zielgruppen

Die Existenz evaluierter Präventionsmaßnahmen führt nicht automatisch zu einer Inanspruchnahme durch die Zielgruppe. Die Inanspruchnahme setzt eine adäquate Ansprache und eine erfolgreiche Motivierung der Zielgruppe voraus, durch welche die intendierten Zielgruppen erreicht werden [46]. In diesem Zusammenhang stellt die Ansprache von Menschen, die sich nicht eigenverantwortlich um ihre Gesundheit kümmern können, eine besondere Herausforderung dar.

Die Ansprache der Zielgruppen kann über folgende Institutionen und Akteur*innen, aber auch soziale Medien erfolgen:

- Professionelle Leistungserbringer im Gesundheitswesen:
  - Haus- oder Fachärzt*innen,
  - Klinikambulanzen (Notaufnahme),
  - Kliniken und Reha-Einrichtungen,
  - Apotheken,
  - ambulante Pflegedienste und Tagespflege,
  - Wohlfahrtsverbände, z. B. Deutsches Rotes Kreuz, Caritas, Diakonie etc.,
  - Bewegungstherapeut*innen,
  - Einrichtungen des betreuten Wohnens,
  - andere.
- Kostenträger im Gesundheitswesen:
  - Krankenkassen,
  - Pflegekassen.
- Sonstige, potenzielle Kontakte mit Personen der Zielgruppe:
  - Kommune, Gesundheitsämter, Bürgerbüros, Quartiersmanager*innen,
  - kirchliche Einrichtungen,
  - Senioren- und Gesundheitsorganisationen,
  - Sportvereine,
  - Vereine/Organisationen, die im ländlichen Raum gut vertreten sind (z. B. Landfrauenverbände).
- Soziale Medien (bspw. Facebook, Twitter, Newsletter).

Es werden vielfältige Arten von Ansprachen durch unterschiedliche Institutionen, Medien und Akteur*innen auch außerhalb des Gesundheitssektors als zielführend angesehen, um sturzgefährdete Personen über Sturzpräventionsprogramme zu informieren, mit dem Ziel, ihnen diese zugänglich zu machen [24, 39]. Neben der Ansprache ist die erfolgreiche Identifikation sturzgefährdeter Personen durch Akteur*innen mit direktem Kontakt eine notwendige Voraussetzung für die Eingliederung der Zielgruppe in entsprechende Sturzpräventionsprogramme. In diesem Zusammenhang kommt Ärzt*innen und anderem Gesundheitspersonal erwartungsgemäß eine entscheidende Rolle zu, da sie regelmäßigen und langfristigen Kontakt zu diesen Personen haben und eine fachliche Einschätzung vornehmen können [7, 38]. Darüber hinaus steigt die Wahrscheinlichkeit einer Assessment- und Programmteilnahme, wenn dies von behandelnden Ärzt*innen und Therapeut*innen empfohlen wird [30, 38]. Die erfolgreiche Identifikation sturzgefährdeter Personen könnte von den Kostenträgern mittels ökonomischer Anreize für die professionellen Leistungserbringer in Form von Bonussystemen optimiert werden. Der Einsatz präventiver Hausbesuche ist noch nicht flächendeckend gewährleistet, könnte jedoch mittelfristig aufgrund seiner Berücksichtigung im aktuellen Koalitionsvertrag an Bedeutung gewinnen. Ein Vorteil wäre, dass Senior*innen in ihrem Zuhause erreicht werden, weshalb das potenzielle Sturzrisiko besser eingeschätzt werden könnte.

Um eine möglichst hohe Bereitschaft zur Teilnahme an Sturzpräventionsprogrammen zu erreichen, muss die übergeordnete Gruppe älterer Personen im ersten Schritt generell darüber aufgeklärt werden, dass regelmäßiges körperliches Training zu einer Reduktion von Stürzen führen kann [46]. Eine defizitorientierte Darstellung, bei der mögliche negative Auswirkungen durch Stürze dargestellt werden, führt zu einer Verringerung der Teilnahmebereitschaft [46, 47]. Entsprechend sollten Programme auch positiv konnotierte Namen tragen. Auch der soziale Aspekt eines Gruppentrainings stellt ein wichtiges Motiv zur Teilnahme dar, welches Berücksichtigung finden sollte [41].

## 7. Etablierte Sturzpräventionsprogramme

In Deutschland existieren bereits einige gruppenbasierte Sturzpräventionsprogramme, die z. T. empirisch überprüft wurden bzw. werden (Übersicht dazu in Tab. 2). Die BIS hat sich mit den meisten Programmen beschäftigt und die Erfahrungen dieser Programme in die vorliegenden Empfehlungen eingearbeitet. In dem umfassenden Review von Sherrington et al. [37] wird grundsätzlich unterschieden nach:

- Krafttraining;
- Flexibilitätstraining;
- dreidimensionalem Training wie Tai-Chi und Tanzformen;
- körperlichem Training jedweder Art; dies beinhaltet Gehtraining, Gleichgewichtstraining, Koordinationstraining und funktionelles Training;
- allgemeiner körperlicher Aktivität als Intervention;
- Ausdauertraining;
- Trainingsformen, welche mehrere der obig aufgeführten Kategorien beinhalten.

**Table 2:** 

Programm	Quellen	Interventionsziele und -inhalte	Evaluation
Sturzprävention	Landessportbünde	Training der Kraft‑, Gleichgewichts- bzw. Koordinationsfähigkeit, mit starkem Alltagsbezug, Bewegungsaufgaben für den Alltag zur Förderung der Selbstständigkeit und langfristigen Reduzierung des Sturzrisikos	Das Programm bezieht sich auf die wissenschaftlichen Empfehlungen des American College of Sports Medicine (ACSM). Evidenz zu Programminhalten und Methoden, Einheiten etc. (Income-Evidenz) liegt vor, v. a. durch Bezugnahme auf das Empfehlungspapier der BIS und die Begutachtung durch den DOSB
Standfest im Alter	Freiberger et al. (2012) [8]	Gleichgewicht/Koordination, Kraft und Gangschulung, Dual- und Multi-Tasking-Training, Trainings von Alltagssituationen, Wissensbausteine und Hausaufgaben, Verhaltensstrategien bei Angst vor Stürzen, Selbstwirksamkeit, Förderung von und Bindung an körperl. Aktivität	Evaluation der verschiedenen Komponenten zeigte die Effektivität insbesondere der körperlichen Trainingsinhalte auf die motorische Leistungsfähigkeit
Standfest und Stabil	Winkler und Regelin (2012) [43]	Kraft- und Gleichgewichtstraining, Dual- und Multi-Tasking-Training, Trainings von Alltagssituationen, Wissensbausteine und Hausaufgaben. Unterstützung von Verhaltensänderung und Selbstbefähigung	Income-Evidenz (s. oben) liegt vor; keine weitere Evaluation
Standfest im Alltag	Zentrum für Altersmedizin Radeburg	Schwerpunkt progressive Gleichgewichts- und Kraftübungen	Evaluation im Pilotprojekt, baut auf „Standfest im Alter“ auf
Trittsicher durchs Leben^a^	DTB/SVLFG/dlv	Training der Kraft‑, Gleichgewichts- bzw. Koordinationsfähigkeit mit starkem Alltagsbezug, Einsatz von Gewichtsmanschetten, Einsatz einer Heimtrainingsbroschüre für ein zusätzliches Training in der häuslichen Umgebung zur Förderung der Selbstständigkeit und langfristigen Reduzierung des Sturzrisikos	Das Trainingsprogramm basiert auf dem Otago-Programm und dem Programm Standfest und Stabil, ist multifaktoriell angelegt und enthält auch Umgebungsanpassung und medikamentöse Maßnahmen zur Osteoporosetherapie. Es wurde im Rahmen einer clusterrandomisierten Studie mit 10.000 Interventionsteilnehmer*innen evaluiert

^a^Als Ganzkörpertrainingsprogramm und *nicht* als reines Gruppentrainingsprogramm zur Sturzprävention mit SPORT PRO GESUNDHEIT und dem Deutschen Standard Prävention zertifiziert

### Effektivität von Einzel- vs. Gruppentrainingsformen

Bei Betrachtung der Effektivität körperlichen Trainings im Allgemeinen zeigte sich ein positiver Effekt im Sinne einer Reduktion der Sturzrate um 23 % im Gruppentraining. Es wurde zudem kein Unterschied in der Effektivität zwischen Einzel- und Gruppentraining nachgewiesen [37, 44]. Gleiches galt bei Betrachtung des Endpunktes „Anzahl der Personen, die einen oder mehrere Stürze erlebten“. Unter Hinzunahme von Krafttrainingsinhalten stieg die Sturzreduktionsrate auf 34 % im Vergleich zu Kontrollgruppen. Auch hier ergab sich kein signifikanter Unterschied zwischen den beiden Darbietungsformen.

Gleichgewichts- und funktionelles Training zogen eine Sturzreduktion von 24 % nach sich, wobei ebenfalls kein Unterschied zwischen den beiden Darbietungsformen sichtbar war.

Tai-Chi als in der Regel im Gruppenformat durchgeführtes Programm zeigte zwar eine Reduktion der Sturzrate um 19 % im Vergleich zu Kontrollgruppen, jedoch wurde aufgrund der zum Teil niedrigen Qualität der Studien der Evidenzgrad als niedrig eingestuft. Die Anzahl der Personen, die einen oder mehrere Stürze erlebten, reduzierte sich um 20 %.

Trainingsformen, welche aus mehreren Komponenten bestehen (meist Gleichgewichtstraining plus funktionelle Übungen plus Krafttraining), zeigten eine Sturzreduktion von 34 % in den Trainingsgruppen im Vergleich zu Kontrollgruppen. Unterschiede der Effektivität zwischen Gruppensetting und individuellem Training zeigten sich nicht. Die Anzahl der Stürzenden reduzierte sich um 22 % durch diese Maßnahmen. Auch hier bestand kein Unterschied zwischen Gruppen- und Einzeltraining.

Studien, welche verschiedene Trainingsformen miteinander verglichen, zeigten komplexere und weniger eindeutige Ergebnisse. Einige Studien fanden keine Unterschiede zwischen Trainingsformen hinsichtlich der Effekte auf die verschiedenen Sturzendpunkte [37]. Allerdings wurde ein Großteil der Studien auch nicht mit angemessen großen Stichproben durchgeführt, sodass die Evidenz hier unklar bleibt. In Metaanalysen zeigt sich eine Dosis-Wirkung-Beziehung in der Form, dass höhere Intensität, häufigere Durchführung und höhere Komplexität zu größeren Effekten führten.

Nur wenige Studien haben ähnliche oder gleiche Trainingsformen hinsichtlich der Darbietungsform (Gruppe vs. individuelles Training) und deren Effekt auf die Sturzrate verglichen. Weder in Bezug auf die Sturzrate, die Anzahl der Personen, die stürzten, noch auf gesundheitsbezogene Lebensqualität oder die Anzahl der Todesfälle zeigt sich ein klares Bild, anhand dessen man eine Empfehlung geben könnte. Die Stichproben der Studien sind nicht von ausreichender Größe.

Eine ausführliche Zusammenfassung der Ergebnisse des Reviews von Sherrington et al. liefern Heupel-Reuter et al. [17].

### Gruppentraining zur Sturzprävention

Seit 2009 wurden verschiedene Programme in Deutschland neu angeboten und evaluiert. Die folgenden Programme werden von der BIS zur Teilnahme empfohlen.

- *Sturzprävention*: Landessportbünde; zertifiziert mit SPORT PRO GESUNDHEIT und dem Deutschen Standard Prävention,
- *Standfest im Alter*: DVGS; zertifiziert mit dem Deutschen Standard Prävention,
- *Standfest und Stabil*: DTB; zertifiziert mit SPORT PRO GESUNDHEIT/Pluspunkt Gesundheit und dem Deutschen Standard Prävention,
- *Standfest im Alltag*: Geriatrie Radeburg; zertifiziert mit dem Deutschen Standard Prävention.

### Multifaktorielles Programm

- *Trittsicher durchs Leben*: SVLFG/dlv/DTB, zertifiziert als Ganzkörperkräftigungsprogramm mit SPORT PRO GESUNDHEIT/Pluspunkt Gesundheit und dem Deutschen Standard Prävention.

## 8. Qualitätssicherung

Qualitätsmanagement in der Sturzprävention ist ein wichtiges Thema, da zunehmend Maßnahmen angeboten werden, die nicht evidenzbasiert sind. Die Sicherung der Qualität dient sowohl dem Kostenträger als auch der Zielgruppe. Sie umfasst eine differenzierte Leistungsbeschreibung und die Festlegung verbindlicher Qualitätsmerkmale und -ebenen (Zieldefinition, Struktur‑, Prozess- und Ergebnisqualität) sowie Maßnahmen zur Qualitätsüberprüfung [3]. Sie dienen als Beurteilungsmaßstab für den Erfolg einer Maßnahme und bilden dann wiederum die Grundlage zur Optimierung der Programme. Zur Überprüfung bedarf es adäquater Instrumente der Diagnostik. In der Sturzprävention kann die Diagnostik auf allen Qualitätsebenen eingesetzt werden. Wesentliche Kriterien zu Struktur‑, Konzept- und Planungs‑, Prozess- und Ergebnisqualität sind dem Leitfaden Prävention zu entnehmen [12].

Die „Zentrale Prüfstelle Prävention“ (ZPP) gilt als zentrales und standardisiertes Prüfsystem zur Qualitätssicherung von Präventionskursen. Gruppentrainingsprogramme werden hierbei nach dem aktuellen Leitfaden Prävention des GKV Spitzenverbandes evaluiert und bei Erfüllung der Anforderungen gemäß § 20 SGB V mit dem Prüfsiegel „Deutscher Standard Prävention“ für eine Zertifizierungsdauer von 3 Jahren ausgezeichnet [48].

Zudem gibt es die Möglichkeit einer Selbstevaluation hinsichtlich der Ergebnisqualität des jeweiligen Kursangebots zu Prävention und Gesundheitsförderung. Diese ist dem Leitfaden *Selbstevaluation für Praktikerinnen und Praktiker* des Landeszentrums Gesundheit Nordrhein-Westfalen zu entnehmen [21].

Für Sturzpräventionsprogramme als Gruppenangebote (vgl. Leitfaden Prävention unter https://www.gkv-spitzenverband.de/krankenversicherung/praevention_selbsthilfe_beratung/praevention_und_bgf/leitfaden_praevention/leitfaden_praevention.jsp) sind die wesentlichen Punkte:

- Alle Maßnahmen sollten effektiv sein (Ergebnisqualität).
- Die Ausführung muss durch Anbieter*innen mit geeigneter fachlicher und pädagogischer Qualifikation (Strukturqualität) erfolgen. Die Qualität der Anbieter*innen von Sturzpräventionsmaßnahmen sollte durch einheitliche Ausbildungs- und Prüfungskriterien im Rahmen der Qualifikation gesichert sein.
- Die Konzepte sollen erprobt, evaluiert (Konzept- und Planungsqualität) und unter angemessenen organisatorischen Durchführungsbedingungen (Prozessqualität) zu erbringen sein.
- Die Maßnahmen sollen möglichst niedrigschwellig angeboten werden, um auch sozial benachteiligte Gruppen zu erreichen.

## 9. Zu Qualifikation und Ausbildung der Kurstrainer*innen

Die Anzahl der sturzgefährdeten Menschen wird in den nächsten Jahren und Jahrzehnten aufgrund der demografischen Entwicklung weiterhin ansteigen. Um die hohe Anzahl an betroffenen Menschen im Handlungsfeld Bewegungsgewohnheiten betreuen zu können, ist die Aus‑, Fort- und Weiterbildung (gem. Berufsbildungsgesetz (BBiG), § 53) von einer ausreichenden Anzahl fachlich und sozial kompetenter Bewegungsfachkräfte erforderlich (vgl. Vorgaben des Deutschen Qualitätsrahmens (DQR) und des Europäischen Qualitätsrahmens (EQR)). Im Sinne des Durchlässigkeitsprinzips des DQR, dem auch die gesetzliche Krankenkasse (GKV) im aktuellen Leitfaden Prävention folgt, sind neben den formal qualifizierten Ausbildungsberufen künftig auch nichtformal qualifizierte Ausbildungsberufe zur Durchführung von sturzpräventiven Bewegungsprogrammen des Präventionsprinzips „Vorbeugung und Reduzierung spezieller gesundheitlicher Risiken durch geeignete verhaltens- und gesundheitsorientierte Bewegungsprogramme“ zugelassen.

Grundsätzlich sollten Kurstrainer*innen über folgende Basisqualifikationen für die Durchführung von Sturzpräventionsprogrammen verfügen: staatlich anerkannter Berufs- und/oder Studienabschluss im Bereich Bewegung, insbesondere Sportwissenschaften (Diplom, Staatsexamen, Magister, Master, Bachelor), Physiotherapie, Sport- und Gymnastiklehrer*in und DOSB-Übungsleiter*in B „Sport in der Prävention“ für die Zielgruppe „Erwachsene/Ältere“ mit der Anerkennung des Qualitätssiegels „SPORT PRO GESUNDHEIT“. Zu unterscheiden sind formal qualifizierte Berufs- und Studienabschlüsse und nichtformal berufliche Qualifizierungen mit Abschluss. Grundlage ist dabei der Nachweis von den im Leitfaden Prävention beschriebenen Mindeststandards in Bezug auf fachwissenschaftliche, fachpraktische und fachübergreifende Kompetenzen für das Handlungsfeld der Sturzprävention.

### Formal qualifizierte Berufs- und Studienabschlüsse

Zu den formalen Qualifikationen zählen die Berufs- und Studienabschlüsse, welche die unten aufgeführten Fachkompetenzen zu mind. 60 % in staatlich anerkannten Berufsausbildungen und/oder wissenschaftlichen Studiengängen an Universitäten oder Fachhochschulen mit Abschluss erworben haben. Diese können bis zu 40 % durch weitere Qualifizierungsmaßnahmen von staatlich anerkannten Institutionen der Aus‑, Fort- und Weiterbildung sowie von Berufs- und Fachverbänden ergänzt werden.

Fachwissenschaftliche Kompetenzen:

1. Trainings- und Bewegungswissenschaften (≥180 h oder 6 ECTS):
  Grundlagen der Bewegungs- und Trainingswissenschaft, Biomechanik,
  Trainingsmethoden, Trainingsinterventionen,
  Trainingsplanung und Belastungssteuerung,
  gesundheitsorientierte Trainingsprogramme planen und durchführen.
2. Medizin (≥150 h oder 12 ECTS):
  funktionelle Anatomie, Physiologie,
  Wirkung von Bewegungs- und Leistungsfähigkeit und Prävention chronischer Erkrankungen.
3. Pädagogik/Psychologie (≥180 h oder 6 ECTS):
  geistes-, verhaltens- und sozialwissenschaftliche Kenntnisse,
  theoretische Grundlagen in Pädagogik und Psychologie/Gesundheitspsychologie.
4. Pathologie/Pathophysiologie (≥120 h oder 4 ECTS):
  Grundlagen der Pathophysiologie, Krankheits- und Schadensbilder, chronische Erkrankungen,
  Indikationen/Kontraindikationen körperlicher Aktivität,
  körperliche Aktivität im rehabilitativen Kontext.

Fachpraktische Kompetenzen:

5. Theorie und Praxis der Sportarten/Bewegungsfelder (≥360 h oder 12 ECTS ausschließlich in Präsenz inkl. Lehrproben):
  Kraftsportarten, Ausdauersportarten, Sportspiele,
  sportspezifische Techniken (z. B. Schwimmen, Nordic-Walking, Aquafitness).

Fachübergreifende Kompetenzen:

6. Grundlagen der Gesundheitsförderung und Prävention (≥180 h oder 6 ECTS):
  Grundlagen der Prävention (primär/sekundär/tertiär) in Lebensphasen und Lebenswelten,
  Verhaltensinterventionen im Gesundheitssport.

### Nichtformale berufliche Qualifizierung mit Abschluss (mit ZPP-Anerkennung)

Für Bewegungsangebote der Sturzprävention können die unter „formal qualifizierte Berufs- und Studienabschlüsse“ beschriebenen Fachkompetenzen auch in einer nichtformalen beruflichen Qualifizierung mit einer Mindestdauer von einem Jahr erworben werden. Die Ausbildung muss mit einer Prüfung abgeschlossen und nachgewiesen werden (vgl. Leitfaden Prävention, 2018, S. 68).

Unter „nichtformal qualifizierte Abschlüsse“ fallen Ausbildungen an privaten Institutionen, welche nicht über das BBiG oder andere spezifische Landesgesetze geregelt sind. Darunter fallen auch die DOSB-Lizenzen „Sport in der Prävention“. Für DOSB-Übungsleiter*innen B „Sport in der Prävention“ für die Zielgruppe „Erwachsene/Ältere“ bieten die Landessportbünde (LSB) und der Deutsche Turner-Bund (DTB) mit seinen Landesturnverbänden (LTV) verschiedene nichtformale Qualifizierungsmöglichkeiten an. Integraler Bestandteil dieser Qualifizierungsmaßnahmen sind die Präventionsprogramme „Sturzprävention“ der LSB und das Sturzpräventionsprogramm „Standfest und Stabil“ des DTB. Voraussetzung für die Anerkennung durch die ZPP ist für diese Gruppe das Qualitätssiegel „SPORT PRO GESUNDHEIT“.

### Nichtformale berufliche Qualifizierung mit Abschluss (ohne ZPP-Anerkennung)

Neben den von der ZPP zertifizierten individualisierten Leistungen der Prävention existieren für Ältere oder Hochaltrige Sturzpräventionsprogramme, die nicht ZPP-zertifiziert werden müssen, wie beispielsweise im Pflegeheim oder in der Kommune. Die Programme müssen aber sinnvoll in das Gesamtkonzept eingebettet werden. Für die Durchführung dieser Bewegungsprogramme können ehrenamtliche Fachkräfte, wie Übungsleiter*innen des Deutschen Roten Kreuzes (DRK) oder Übungsleiter*innen C bzw. Trainer*innen C „Fitness und Gesundheit“ des Deutschen Olympischen Sportbundes (DOSB), eingesetzt werden.

## 10. Finanzierung

Eine flächendeckende Verbreitung und Umsetzung von Programmen zur Sturzprävention werfen eine Reihe von Fragen zur Finanzierung auf. Neben einer Darstellung der Kostenstruktur von Programmen werden in diesem Kapitel die gesetzlichen Grundlagen dargestellt, die bei einer Finanzierung durch die gesetzlichen Kostenträger (gesetzliche Krankenversicherungen) berücksichtigt werden müssen.

### Kostenstruktur

Finanzielle Aufwände entstehen sowohl durch die Verbreitung als auch durch die praktische Umsetzung eines Sturzpräventionsprogramms:

- Verbreitung:
  - Kosten für Medienkampagnen.
- Praktische Umsetzung
  - Ausbildung und Honorierung der Kursleiter*innen,
  - Transportkosten (Fahrt der Teilnehmer*innen zu den Angeboten),
  - Organisationskosten (Verwaltung der Angebote, Räume, Material, Geräte).

### Gesetzliche Grundlagen zur Finanzierung durch gesetzliche Kostenträger

Die Möglichkeit zur finanziellen Förderung von Primärpräventionsleistungen durch gesetzliche Krankenversicherungen ist durch *§ 20 SGB V Primäre Prävention und Gesundheitsförderung *gegeben. Die Prävention von Stürzen wird in *§ 20 SGB V* nicht ausdrücklich als Handlungsfeld der Primärprävention genannt. Dennoch kann die Förderung von Sturzpräventionsprogrammen gesetzeskonform und möglich sein, da nach *§ 20 Abs. 4 Nr. 1 SGB V* die „Vorbeugung und Reduzierung spezieller gesundheitlicher Risiken durch geeignete verhaltens- und gesundheitsorientierte Bewegungsprogramme“ ein Handlungsfeld der „individuellen verhaltensbezogenen Primärprävention“ darstellt. Dementsprechend werden in dem *Leitfaden Prävention* zertifizierte „Trainings zur Sturzprävention“ ausdrücklich als förderfähige Maßnahmen i. S. des *§ 20 Abs. 4 Nr. 1 SGB V* dargestellt. Die bisher regional durchgeführten Projekte und Maßnahmen der Sturzprävention wurden seitdem von den gesetzlichen Krankenkassen und weiteren, häufig regionalen Förderern getragen. Konkrete Projekte, Programme etc. müssen entwickelt, evaluiert und gemäß § 20 Absatz 2 Satz 2 SGB V zertifiziert werden, um eine Finanzierung durch die gesetzlichen Krankenkassen zu ermöglichen.

Nach *§ 20 Absatz 6 SGB V* sollen gesetzliche Krankenversicherungen pro Jahr und pro Versicherten in Summe mindestens 2,20 € (Stand 2020) in Leistungen nach *§ 20a SGB V Leistungen zur Gesundheitsförderung und Prävention in Lebenswelten* (z. B. Maßnahmen im Setting Kommune) investieren. Seit dem 25.07.2015 sind die Krankenkassen zu diesen Ausgaben für Leistungen nach *§ 20a SGB V *verpflichtet. Zusätzlich sind Leistungen* zur Prävention in stationären Pflegeeinrichtungen nach § SGB XI *(Pflegleistungen) denkbar, wenn sie in ein Projekt in der Einrichtung eingebunden sind. Die Kassen stellen hierfür 0,33 € (2020) pro Versicherten zur Verfügung. Unterschreiten die jährlichen Ausgaben einer gesetzlichen Krankenversicherung diese Vorgabe, so muss die Krankenkasse die nichtausgegebenen Mittel im Folgejahr für Leistungen nach *§ 20a SGB V* oder § 5 SGB XI zur Verfügung stellen. Diese Gesetzesänderung stellte eine deutliche Stärkung der Finanzierung der Primärpräventionsmaßnahmen dar. Zudem besteht die Verpflichtung, die Vorgabe der Mindestausgaben für Präventionsleistungen jährlich gemäß *§ 18 SGB IV* anzupassen, was in etwa zu einer Anpassung der Mindestausgaben entsprechend der Inflationsrate führt. Für 2020 wurden diese Pflichtausgaben allerdings wegen der Corona-Krise ausgesetzt.

Des Weiteren ist eine anteilige Kostenerstattung auch aus den folgenden Leistungsbereichen denkbar:

- Präventionsprojekte auf Landesebene im Rahmen der Landesvereinbarungen zur Prävention und Gesundheitsförderung nach § 20 f SGB V (z. B. Projekte in der Lebenswelt-Kommune),
- ergänzende Leistungen zur Rehabilitation, z. B. Patient*innenschulung nach § 43 Abs. 1 Nr. 2 SGB V bei Vorliegen einer ärztlichen Empfehlung („Maßnahmen zur Verhütung von Verschlimmerung von Erkrankungen, tertiäre Prävention“),
- Rehabilitationssport nach § 64 Abs. 1 Nr. 3 oder Funktionstraining nach § 64 Abs. 1 Nr. 4 (Zielgruppe „behinderte oder von Behinderung bedrohte Menschen“),
- im Rahmen von befristeten Modellvorhaben nach § 20 g SGB V (zur Prävention und zur Gesundheitsförderung) mit wissenschaftlicher Begleitung und Auswertung.

Die Vermeidung von Stürzen und deren Folgen für die Gesellschaft und die Gesundheitskosten ist aber letztlich eine gesamtgesellschaftliche Aufgabe und kann nicht ausschließlich mit einer Bezuschussung durch die Krankenkassen dauerhaft durchgeführt werden. Hier sind Bund, Länder und Gemeinden (z. B. im Rahmen des öffentlichen Gesundheitsdienstes, der Bauordnung und der allgemeinen Flächenplanung) genauso gefordert, wie private Krankenkassen und die Pflegekassen sowie die Bürger*innen selbst.
